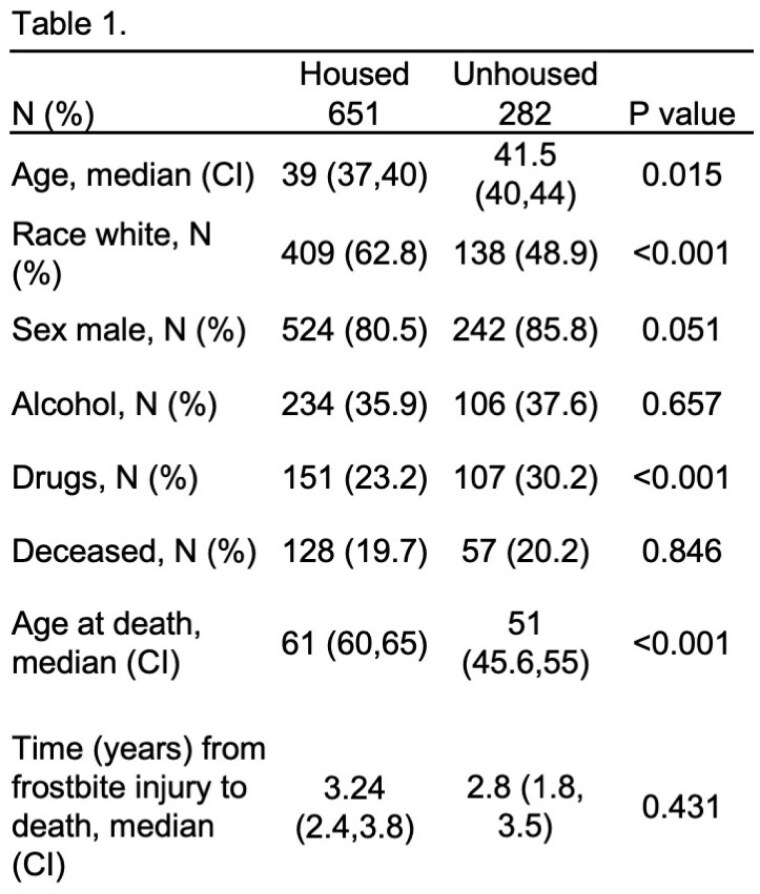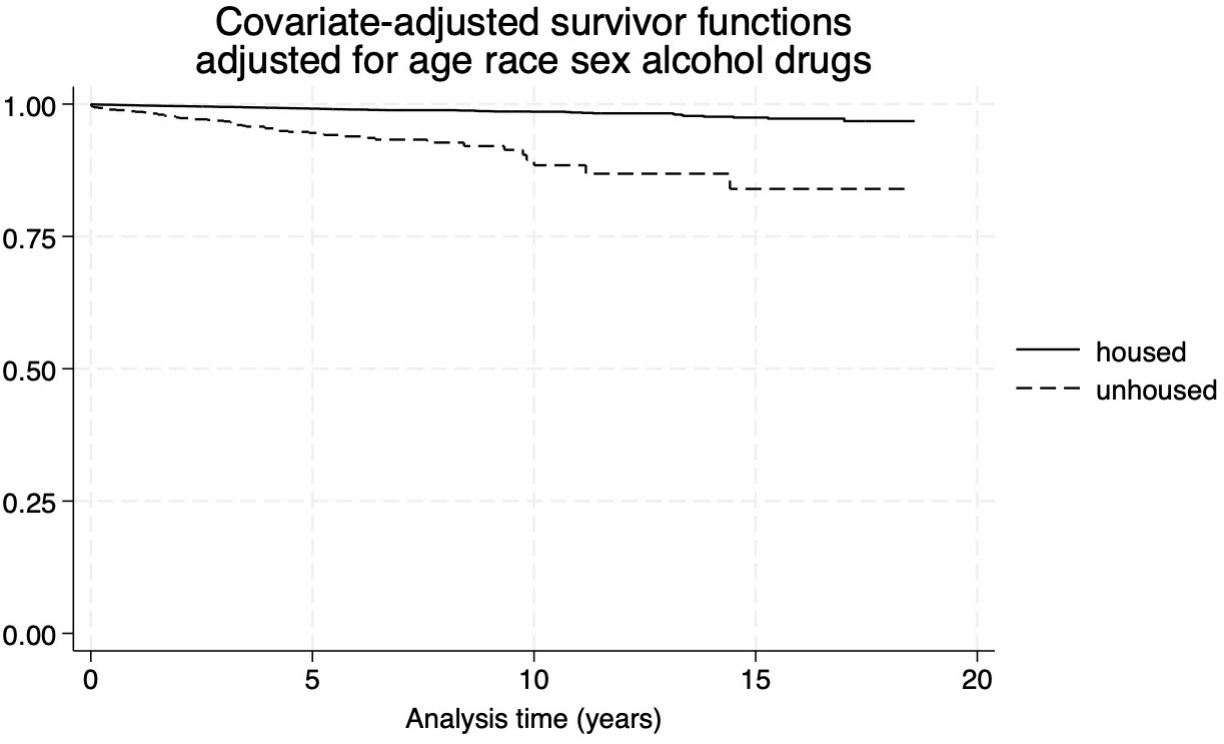# 15 Impact of Housing Status on Mortality Following Frostbite Injury: A Multi-Center Longitudinal Cohort Analysis

**DOI:** 10.1093/jbcr/iraf019.015

**Published:** 2025-04-01

**Authors:** Alexandra Lacey, Emily Colonna, Rediat Tilahun, Rachel Nygaard

**Affiliations:** Regions Hospital Burn Center; Hennepin Healthcare; Hennepin Healthcare Research Institute; Hennepin Healthcare

## Abstract

**Introduction:**

Many factors contribute increased risk of frostbite injury including housing status, psychiatric illness, substance abuse history, and age. We hypothesized that these individual factors may increase the risk of mortality within this patient population. To evaluate this, we analyzed 15 years of longitudinal data from two high volume frostbite centers.

**Methods:**

Burn registries were abstracted for frostbite injured patients that survived their initial hospital stay with follow-up data obtained via linkage to state health department records. A limited data set was shared and analyzed for factors associated with long-term mortality following frostbite injury. Cox proportional hazards regression evaluated the impact of housing status on mortality and was adjusted for age, sex, race, alcohol use, and drug use.

**Results:**

This longitudinal cohort study examined 925 frostbite patients with nearly one third of these patients lacking stable housing at the time of diagnosis. Table 1 details other patient characteristics. At the end of the study period, we found that 185 patients were deceased (19.8%). Time from frostbite injury to death ranged from 0.014 years to 17 years. Similar proportions of unhoused and housed frostbite patients died in the follow-up period (19.7% vs 20.2%). The proportion of sex, race, alcohol, and drugs did not differ significantly between those that died during the follow-up period. The age of death was significantly lower in persons lacking stable housing. Significantly higher proportions of unhoused persons were non-white race. Age was a significant predictor of mortality, while sex, race, and alcohol were not. Living situation and drug use showed a trend toward increased mortality risk, but this did not reach significance. The model (LR chi² = 150.43, p < 0.001) indicated that the combined set of predictors significantly explained the variability in mortality risk (fig 1).

**Conclusions:**

This is the first multicenter study to examine long-term mortality risk of frostbite-injured patients. By using a large cohort and state health records, it offers new insights into socioeconomic factors influencing survival. Unhoused persons are at higher risk for poor outcomes following traumatic injury and require additional resources. These findings emphasize the need for tailored healthcare strategies and resource allocation to reduce mortality in this vulnerable population.

**Applicability of Research to Practice:**

These findings highlight the influence of age on mortality, while suggesting trends for socioeconomic and behavioral factors that warrant further investigation.

**Funding for the Study:**

N/A